# Functional dyadicity and heterophilicity of gene-gene interactions in statistical epistasis networks

**DOI:** 10.1186/s13040-015-0062-4

**Published:** 2015-12-21

**Authors:** Ting Hu, Angeline S. Andrew, Margaret R. Karagas, Jason H. Moore

**Affiliations:** 1 Department of Computer Science, Memorial University, St. John’s, NL, Canada; 2 Department of Community and Family Medicine, Geisel School of Medicine, Dartmouth College, Hanover, NH USA; 3 Department of Biostatistics and Epidemiology, The Perelman School of Medicine, University of Pennsylvania, Philadelphia, PA USA

**Keywords:** Dyadicity, Heterophilicity, Epistasis, Gene-gene interactions, Genetic association studies, Statistical epistasis networks, Functional annotation, Bladder cancer, DNA repair, Cholesterol and sterol transport

## Abstract

**Background:**

The interaction effect among multiple genetic factors, i.e. epistasis, plays an important role in explaining susceptibility on common human diseases and phenotypic traits. The uncertainty over the number of genetic attributes involved in interactions poses great challenges in genetic association studies and calls for advanced bioinformatics methodologies. Network science has gained popularity in modeling genetic interactions thanks to its structural characterization of large numbers of entities and their complex relationships. However, little has been done on functionally interpreting statistically inferred epistatic interactions using networks.

**Results:**

In this study, we propose to characterize gene functional properties in the context of interaction network structure. We used Gene Ontology (GO) to functionally annotate genes as vertices in a statistical epistasis network, and quantitatively characterize the correlation between the distribution of gene functional properties and the network structure by measuring dyadicity and heterophilicity of each functional category in the network. These two parameters quantify whether genetic interactions tend to occur more frequently for genes from the same functional category, i.e. dyadic effect, or more frequently for genes from across different functional categories, i.e. heterophilic effect.

**Conclusions:**

By applying this framework to a population-based bladder cancer dataset, we were able to identify several GO categories that have significant dyadicity or heterophilicity associated with bladder cancer susceptibility. Thus, our informatics framework suggests a new methodology for embedding functional analysis in network modeling of statistical epistasis in genetic association studies.

## Background

The goal of genetic association studies is to identify heritable genetic factors that can help explain common human diseases and phenotypic traits [1–3]. Recent rapid development of sequencing technologies enables genotyping thousands to millions of single nucleotide polymorphisms (SNPs) for testing their phenotypic associations and thus brings the genetic association studies to a new era [4, 5]. Although studies have uncovered numerous disease susceptibility loci over the years [1, 6, 7], the majority of them were only able to find limited associations between individual genetic factors and disease risks with commonly used main-effect based methods [8]. The non-linear interaction effect among multiple genetic attributes has been realized to play an important role explaining the missing heritability [9, 10]. This interaction effect, also defined as *epistasis*, describes the departure of independence among multiple genetic attributes associated with a particular phenotypic outcome [11–14]. Epistasis holds great potentials and has become a new focus of genetic association studies [15–17]. However, it also poses great statistical and computational challenges due to the high dimensionality and computational demands of interaction analysis [18, 19].

Network science has gained popularity in biological sciences thanks to its ability of modeling complex relationships among a large number of entities [20, 21]. A network is generally defined by a collection of vertices joined in pairs by edges. It has been used to study biological systems at multiple levels of organization including metabolisms [22], protein-protein interactions [23], genetic regulatory networks [24], and food webs [25]. It also provides a very suitable framework for epistasis studies since it allows for a structural representation of a large number of genetic attributes and their interaction relationships [26]. A number of genetic association studies have used networks to characterize epistatic interactions and have seen successful applications to various human diseases and traits [27–29].

Most existing epistasis network methodologies construct genetic interaction networks by assigning vertices as genetic attributes, e.g. SNPs or genes, and linking pairs of vertices if significant interaction relationships are detected, either biologically or statistically. Then vertices with outstanding network properties are identified as key vertices including hubs, i.e. vertices with a significantly larger number of neighbors than average, or bottlenecks, i.e. vertices with high centralities that hold essential positions on information transmission flows between all pairwise vertices in a network. Annotation of these key vertices is then used to prioritize particular functional categories, such as pathways, with high disease/phenotype association and to propose hypothesis for further biological validations [30–32]. In this study, we take a different route incorporating functional annotation in genetic interaction networks by analyzing the distribution of vertex functional characteristics in the context of network structure.

In most complex networks, besides contributing to the network topology, vertices may possess various characteristics, for instance individual education level in social networks or biological functions in protein-protein interaction networks [24]. The distribution of these vertex characteristics may not be random in the networks but likely correlated with the underlying network structure. There are, in fact, many empirical observations that vertices with similar characteristics tend to be linked together or vice versa [33]. However, not much analytical methodologies have been proposed to quantify such correlations. A recent study by Park et al. [34] proposed using two parameters, dyadicity and heterophilicity, to quantify such interplay between the distribution of vertex properties and the network structure. Their method was applied to complex networks including protein-protein interaction network and mobile service network, and proved effective using these two parameters to quantify the dyadic and heterophilic effects of the distribution of vertex properties.

In this study, we adopted the dyadicity and heterophilicity measurements to characterize gene-gene interactions in the context of epistasis networks. Previously we developed the framework of inferring large scale genetic interactions using Statistical Epistasis Networks (SEN) in disease association studies [27, 35, 36]. We constructed a gene-gene interaction network based on the SEN methodology and investigated the distribution of Gene Ontology functions of genes in such an interaction network. This analysis was expected to help elucidate the varying properties of gene-gene interactions for different functional categories, and thus help us to better understand the underlying biology of statistical genetic interactions.

## Methods

### Bladder cancer dataset

We used a population-based bladder cancer dataset in this study. Bladder cancer cases were from residents of New Hampshire identified in the State Cancer Registry. The cancer patients are of ages 25 to 74 years, diagnosed from July 1, 1994 to June 30, 2001. Healthy controls of age under 65 were selected using population lists from the New Hampshire Department of Transportation, and those of age 65 and above were chosen from data files provided by the Centers for Medicare & Medicaid Services (CMS) of New Hampshire. More than 95 % of the population were of Caucasian origin. Each participant provided informed consent and all data collection procedures and study materials were approved by the Committee for the Protection of Human Subjects at Dartmouth College.

In the genotyping process, DNA was isolated from peripheral circulating blood lymphocyte specimens using Qiagen genomic DNA extraction kits (QIAGEN inc., Valencia, CA). All DNA samples of sufficient concentration were genotyped using the GoldenGate Assay system by Illumina’s Custom Genetic Analysis service (Illumina, Inc., San Diego, CA). Ninety nine point five percent of the submitted samples were successfully genotyped, and samples repeated on multiple plates yielded the same call for 99.9 % of the SNPs. SNPs with more than 5 % missing data were removed from our analysis, and the remaining missing genotypes were imputed using alleles of the highest frequencies across the population. The final dataset includes 1422 SNPs from 396 cancer susceptibility genes from 491 bladder cancer cases and 791 healthy controls. More details of this dataset were discussed in [37, 38].

### Gene interaction network

We previously developed a framework of Statistical Epistasis Networks (SEN) to infer the global structure of interactions among a large set of genetic attributes in genetic association studies [27]. First, all the pairwise epistatic interactions were measured using the information theoretic metric of *information gain* [39, 40]. Specifically, given a pair of SNPs *A* and *B*, the amount of information each of them explains on the phenotypic outcome *C* was measured using *mutual informationI*(*A*;*C*) and *I*(*B*;*C*). When joining *A* and *B*, *I*(*A,B;C*) captured the total association of *A* and *B* together on *C*. Subtracting the individual associations of *I*(*A*;*C*) and *I*(*B*;*C*) from *I*(*A,B;C*), i.e. the information gain *IG*(*A*;*B*;*C*), provided the gain of information on *C* by combining *A* and *B* together, and served as the measure of epistatic interaction between *A* and *B* on *C*.

Then networks were built by including pairs of SNPs as connected vertices if their epistatic interaction strengths were stronger than a theoretically derived threshold. We used global network properties, including the size of the network, the size of the giant connected component and vertex degree distribution, and permutation testing to derive a threshold for including SNP pairs when the network built from the real data showed the most distinguishing topological properties than permuted data networks. Such statistical epistasis networks were able to capture the global interaction structure of a large set of SNPs.

The SEN framework was successfully applied to the population-based bladder cancer dataset, and we were able to identify a SNP interaction network that had a significantly larger giant connected component and a distinguishing heavy-tail degree distribution, compared to all permuted data networks built using the same pairwise interaction threshold [27]. The finding of such a network proposes an important hypothesis of the existence of a large connected structure of complex interactions among bladder cancer associated SNPs, and calls for further validations and investigations.

In the current study, we used Gene Ontology to assign function annotation of each gene and look into the characterization of vertex properties in the epistasis interaction network. Therefore, we built a gene-gene interaction network from the previously identified SNP-SNP interaction network of bladder cancer. In the gene interaction network, each vertex represented a gene, and two genes were connected by an edge if there existed at least one pair of SNPs, one from each gene, that were connected in the identified SNP-SNP interaction network. Transforming the SNP-SNP interaction network to the gene-gene interaction network allowed functional categorizing directly on genes as vertices in the network since the Gene Ontology annotation is on the gene level.

### Gene ontology annotation

We used the Database for Annotation, Visualization and Integrated Discovery (DAVID) [41] to functionally annotate the 185 genes in our epistasis network based on Gene Ontology. The FAT level was used for biological process (BP), cellular component (CC), and molecular function (MF) annotations. GO categories were considered significantly enriched in our network if their enrichment significances were higher than the conventionally used threshold 0.05. We set the gene-in-category count threshold to 3, i.e., we included GO terms in the annotation analysis only if they had at least three genes from our 185 network genes.

### Distribution of vertex properties in networks

Networks have been used to model interactions in complex systems in various areas from biological sciences, engineering, to social science. In most real complex networks, vertices themselves also possess functional characteristics, and observing the distribution of vertex characteristics in the context of network structure provides insights into whether vertices with similar functions tend to connect to each other. A recent study on complex networks [34] proposed a quantitative approach to depicting the interplay between vertex properties and the structure of the underlying network. They proposed two parameters, *dyadicity* and *heterophilicity*, to measure to what degree the vertex characteristics are correlated with the network structure.

**Fig. 1:**
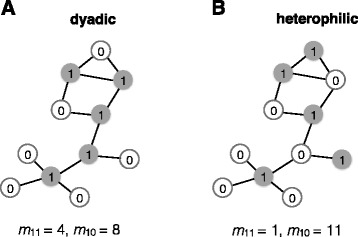
Examples of dyadic and heterophilic distributions of vertex properties in a network. A vertex can either have (value 1) or not have (value 0) a given property. For a given number of vertices with the property (*n*_1_=5 in this example), if there are more similar connections among them, e.g. (1-1 edges), than expected randomly this property is dyadic in the network (**a**), and if there are more connections between vertices with and without the property, e.g. (1-0 edges), than expected randomly the distribution is heterophilic (**b**)

Given a network with known vertex characteristics, dyadicity and heterophilicity can be used to describe the statistical distribution of vertex characteristics in the network. Assuming that each vertex is characterized by a property that takes two values, 1 or 0, in the context of gene interaction networks, a gene contributing to a specific GO term (1) or not (0), *n*_1_ (*n*_0_) denotes the total number of vertices that take value 1 (0) for the given property. In the network, there exist three types of dyads, defined as a pair of vertices and the edge linking them, 1) an edge and its two end vertices that both have value 1, 2) an edge and its two end vertices that take each of the values 1 and 0, and 3) an edge and its two end vertices that both have value 0. The numbers of such three types of dyads in the network are denoted by *m*_11_, *m*_10_, and *m*_00_ respectively. Note that *n*_1_+*n*_0_=*N* and *m*_11_+*m*_10_+*m*_00_=*M*, where *N* is the total number of vertices and *M* is the total number of edges in the network. With a given number *n*_1_, if the property is distributed randomly among *N* vertices, i.e. each vertex has an equal chance of either having or not having such a property, the expected number of (1-1) and (1-0) dyads are

(1) $$\begin{array}{@{}rcl@{}} && \overline{m}_{11} = {n_{1}\choose 2} \times p = \frac{n_{1}(n_{1}-1)}{2}p, \end{array} $$

(2) $$\begin{array}{@{}rcl@{}} && \overline{m}_{10} = {n_{1}\choose 1} {n_{0}\choose 1}\times p = n_{1}(N-n_{1})p, \end{array} $$

where $p=\frac {2M}{N(N-1)}$ calculates the average probability that two vertices are connected. Significant departures from such expected numbers of dyads indicate that the property is not randomly distributed in the network. Therefore, the dyadicity (*D*) and heterophilicty (*H*) can be defined as [34]

(3) $$\begin{array}{@{}rcl@{}} && D= \frac{m_{11}}{\overline{m}_{11}}, \end{array} $$

(4) $$\begin{array}{@{}rcl@{}} && H= \frac{m_{10}}{\overline{m}_{10}}, \end{array} $$

where *m*_11_ and *m*_10_ are observed numbers of dyads in the network. If a significant *D*>1 is observed, the vertex property is dyadic in the network, meaning that more vertices with such a property tend to connect to each other than expected for a random configuration. A significant *H*>1 indicates that the property is heterophilic, meaning that vertices with such a property have more connections to vertices without the property than expected randomly (Fig. 1). Note that it is possible that a node property in a network is both dyadic and heterophilic when nodes with value 1 are mostly hub nodes and are well connected to one another. In this scenario, both the numbers of (1-1) dyads and (1-0) dyads can be significantly greater than null distributions. The significance level of an observed *D* (*H*) can be estimated using permutation testing, where the assignment of vertices’ property values are randomly shuffled while the the total number of vertices taking value 1, i.e. *n*_1_, is fixed. We adopted these two parameters in our study to quantify whether genetic interactions happen more among genes contributing to the same GO functional category or across different functional categories. Also note that the analyses on dyadicity and heterophilicity of different vertex properties, or functional categories, are independent of each other. That is, vertex properties, or functional categories, are not required to be mutually exclusive.

## Results

### Gene interaction network of bladder cancer

**Fig. 2:**
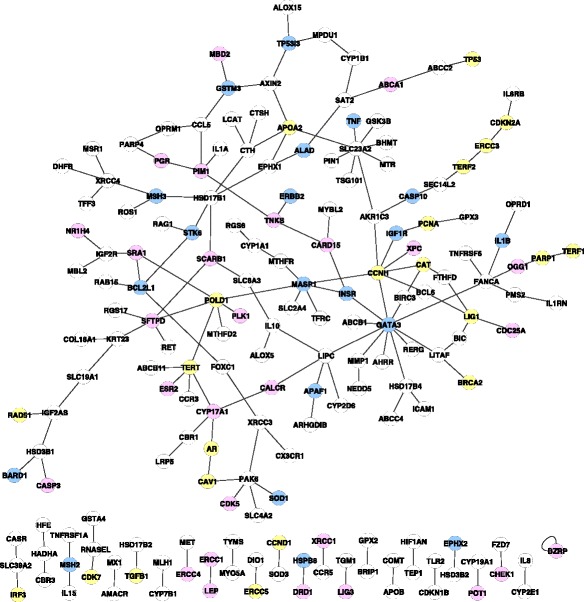
The gene interaction network of bladder cancer. Each vertex represents a gene, and two genes are connected by an edge if there exist at least one pair of SNPs, one from each gene, that have strong and statistically significant interaction associated with bladder cancer and appear as connected vertices in the previously identified statistical epistasis network [27]. The network includes 185 vertices and 174 edges. Colors code for genes mapped to GO categories with significant dyadicity (pink), significant heterophilicity (blue), or both types (yellow). This graph was rendered using Cytoscape [45]

In our previous study on the statistical epistasis network of bladder cancer, we identified a network consisting of 319 SNPs as vertices and 255 edges that had significantly higher connectivity and a more distinguishing degree distribution than expected randomly [27]. In the current study we mapped these 319 SNPs to 185 genes and built a gene-gene interaction network, where each vertex was a gene and two genes were connected if they had at least one pair of underlying SNPs that were connected vertices in the SNP-SNP statistical epistasis network. As shown in Fig. 2, the gene-gene interaction network of bladder cancer had 185 vertices and 174 edges including 1 self-loop. The network was comprised of 25 connected components and the largest connected component included 134 genes. The average number of neighbors of vertices was 1.87.

### Enriched gene functional categories

Gene Ontology annotation using DAVID returned 808 GO terms as significantly enriched functional categories for our set of 185 network genes. The category of the largest gene-in-category count was GO_MF_FAT *nucleotide binding* that had 48 genes, followed by GO_BP_FAT *response to organic substance* (45 genes), GO_CC_FAT *cell fraction* (45 genes), and GO_CC_FAT *membrane-enclosed lumen* (45 genes). We then used these enriched 808 GO terms as vertex properties to perform the analysis on the distribution of vertex characteristics in the network.

### Dyadicity and heterophilicity of enriched GO categories

Each of the enriched GO terms was set as a vertex property, and we assigned each vertex a value of 1 for the property if the represented gene was in the GO category and 0 if not. The dyadicity (*D*) and heterophilicity (*H*) values were then calculated for each of the 808 GO categories. A 100,000-fold permutation test was used to estimate the significance of observed *D* and *H*, by shuffling the assignment of vertex property values. The *p*-value was calculated as the number of *D* (*H*) values of permuted networks that were greater than or equal to the observed values of the real network.

**Fig. 3:**
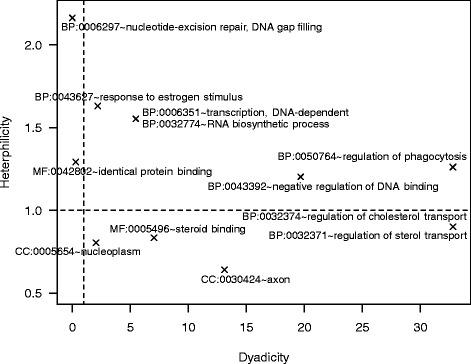
Dyadicity and heterophilicity of enriched and significant GO categories for bladder cancer gene interaction network. The figure includes 12 GO terms that have either significant dyadicity or heterophilicity in the network. Note that two pairs of GO terms have the same dyadicity and heterophilicity values and their data points are on top of each other in the graph. Dashed lines represent *D*=1 and *H*=1, expected from random distributions, for a visual guidance

Table 1 lists the 12 GO categories that had either significant dyadicity or heterophilicity using a *p*-value threshold of 0.05. The number of genes in these categories ranged from 30 (*nucleoplasm*) to 3 (*regulation of phagocytosis*, *nucleotide-excision repair, DNA gap filling*, *regulation of sterol-transport*, and *regulation of cholesterol transport*). See Fig. 2 color coding for genes that mapped to significantly dyadic categories (pink), heterophilic categories (blue), or both categories (yellow). A significant dyadicity indicated that genes from such categories tend to interact more with genes from the same functional categories than expected randomly. The category with the most significant dyadicity was *negative regulation of DNA binding* (*D*=19.676,*p*_*D*_=0.006). Given the structure of the network, it was shown highly significant that two pairs of genes were connected within the total 5 genes in this functional category. A significant heterophilicity, on the opposite, indicated that genes from such categories tend to interact more with genes from different functional groups than random. The most significant heterophilic category was *response to estrogen stimulus* with a *H*=1.630 and a *p*_*H*_=0.015. Figure 3 depicts the graph of the dyadicity and heterophilicity of these 12 significant GO categories. Note that, 8 out of these 12 significant GO terms are from the BP category, including all 5 terms that have significant heterophilicity observations.

**Table 1:** Dyadicity and heterophilicity analysis results of the bladder cancer gene interaction network

Gene Ontology terms	*n*_1_	*m*_11_	*m*_10_	$\overline {m}_{11}$	$\overline {m}_{10}$	*D*	*H*	*p*_*D*_	*p*_*H*_
*Nucleoplasm*	30	9	38	4.422	47.265	2.035	0.804	**0.042**	0.954
*Identical protein binding*	27	1	56	3.568	43.362	0.280	1.291	0.977	**0.026**
*Response to estrogen stimulus*	10	1	29	0.457	17.788	2.186	1.630	0.401	**0.015**
*Transcription, DNA-dependent*	9	2	25	0.366	16.101	5.466	1.553	0.062	**0.036**
*RNA biosynthetic process*	9	2	25	0.366	16.101	5.466	1.553	0.064	**0.036**
*Steroid binding*	8	2	12	0.285	14.393	7.027	0.834	**0.042**	0.776
*Axon*	6	2	7	0.152	10.917	13.118	0.641	**0.013**	0.952
*Negative regulation of DNA binding*	5	2	11	0.102	9.148	19.676	1.202	**0.006**	0.301
*Regulation of phagocytosis*	3	1	7	0.030	5.550	32.794	1.261	**0.045**	0.260
*Nucleotide-excision repair, DNA gap filling*	3	0	12	0.030	5.550	0	2.162	1	**0.037**
*Regulation of sterol-transport*	3	1	5	0.030	5.550	32.794	0.901	**0.047**	0.616
*Regulation of cholesterol transport*	3	1	5	0.030	5.550	32.794	0.901	**0.048**	0.618

A 100,000-fold permutation testing was used to estimate the significance levels of the calculated *D* and *H*, and the *p*-values less than the threshold 0.05 were noted in bold font

## Discussion

In this article, we proposed the methodology of analyzing the distribution of gene functional properties in the context of statistical epistasis networks. The gene interaction network was constructed by first identifying the network of strong and significant pairwise SNP epistatic interactions and then building gene network on top of the SNP interaction network. After annotating genes as vertices based on their functional Gene Ontology, dyadicity and heterophilicity analysis was performed for each GO term to investigate to what degree the vertex characteristics correlate with the underlying interaction network topology. Using a population-based bladder cancer dataset and its previously identified SNP statistical epistasis network, we performed the dyadicity and heterophilicity analysis on enriched GO terms for the genes in the gene interaction network associated with bladder cancer. We were able to find 12 GO categories with significant dyadicity or heterophilicity, which indicated the differential interaction patterns among genes from various functional categories, i.e. some functional categories tend to have genes interacting with each other within the same categories whereas genes from some other functional categories tend to interact more with genes from other categories.

This study complements our previous framework of statistical epistasis networks by constructing gene interaction networks and further analyzing the distribution of gene functional characteristics in the networks. Network science has become very powerful in modeling epistatic interactions in genetic association studies. It is capable of representing and analyzing complex interactions among a large number of genetic attributes. However, less has been done on incorporating functional properties of genetic attributes in the context of interaction networks. Our work analyzed the interplay between functional properties and network topology and provides important insights into the interpretation of the interactions and better understandings of the etiology of the associated diseases.

The bladder cancer gene interaction network had a large connected giant component. This indicates the complex genetic architecture underlying bladder cancer. A total of 808 functional categories were enriched across the 185 genes in the gene interaction network using GO functional annotation analysis. Seven GO terms were significantly dyadic and five others were significantly heterophilic. These different interaction properties of GO categories provide useful insights in understanding various functional components in the etiology of bladder cancer. For instance, note that the functional category *nucleotide-excision repair, DNA gap filling* was enriched in our set of network genes and was shown possessing significantly high heterophilicity (*H*=2.162, *p*_*H*_=0.037). DNA repair genes were previously found to be associated with bladder cancer susceptibility [37]. The current study demonstrates that these genes contribute to bladder cancer susceptibility through epistatic interactions, and their interaction effect is heterophilic, which could indicate that, rather than depending on each other, DNA repair genes would be more likely to interact with genes from other functional categories. SNPs that lead to an increase in the level of DNA damage, (i.e. by increasing the bioactivation of toxins to reactive intermediates), could synergize with impaired DNA repair mechanisms, leading to a greater than additive increase in cancer risk.

Also note that *regulation of cholesterol transport* (*D*=32.794, *p*_*D*_=0.048) and *regulation of sterol transport* (*D*=32.794, *p*_*D*_=0.047) that included genes APOA2, BZRP, and LEP in the network, were enriched and found highly dyadic in the gene interaction network. A growing body of literature suggests increased risk of cancers, including bladder, is associated with high intake of dietary cholesterol [42]. Recent studies have identified the role of cholesterol homeostasis as potential targets for cancer therapeutics [43]. It has been well accepted that excess cholesterol and intermediates of the cholesterol biosynthesis pathway are needed for cancer cells to maintain a high level of proliferation, and the cholesterol and sterol transport mechanisms could be used as potential targets for cancer drug design [44]. Our results suggest that the interaction effects of cholesterol and sterol transport regulation genes, mostly dyadic, contribute to the susceptibility of bladder cancer, and might be useful for future identification of cancer drug targets. We also speculate that the dyadic interaction effect could be the indication that cholesterol transport molecules must bind to cholesterol and to each other to move cholesterol through the body since it is insoluble in blood and many of them exhibit feedback regulation. Therefore regulation of cholesterol and sterol transports have more protein-protein interactions among themselves that are reflected as statistical epistasis interactions in relation to bladder cancer than with other functional groups.

Our methodology itself has great application potential in genetic association studies. It can be used to analyze and interpret the gene-gene interactions for a wide range of phenotypes or diseases. In the current study, we adopted GO annotation with the limitations including that the categorizations are assigned based on current knowledge but many change as new scientific discoveries are made, and that categories are sometimes subsets of one another. In future extensions and applications, we are interested in using other functional annotation methods, such as pathways, drug-, and environment-associations, to look into how these different methods of functional categorization interplay with the vertex property distribution in the gene interaction networks.
